# Predictors of not wanting to seek help or information for suicide thoughts

**DOI:** 10.1186/s12888-025-06985-z

**Published:** 2025-05-26

**Authors:** Christine Mohn, Egil Haga, Hanne Sofie Wernoe Nilsson, Jane Pirkis, Lars Mehlum

**Affiliations:** 1 Institute of Clinical Medicine, National Centre for Suicide Research and Prevention (NSSF), University of Oslo, Kirkeveien 166, Oslo, 0450 Norway; 2 Diakonhjemmet Hospital, Oslo, Norway; 3 Centre for Mental Health, Melbourne School of Population and Global Health, University of Melbourne, Parkville, Australia

**Keywords:** Help-seeking, Mental health, Mental health stigma, Suicide, Suicide prevention

## Abstract

**Background:**

Approximately half of all individuals who die by suicide in Norway do not seek contact with specialist mental health services during the year before death. Here we aim to report the demographic characteristics and attitudes to suicide, mental ill health, and help-seeking in people who do not wish to seek help or information for suicide thoughts. Moreover, we identify the strongest predictors of non-help-seeking intentions.

**Methods:**

A population sample (*N* = 3251) from the Mid- and West- Norway regions participated in an online survey of attitudes to suicide and help-seeking for mental ill health. The sample was recruited from the regional population that later was exposed to a media suicide prevention campaign. Of these, 167 individuals stated that they would not seek help or information if they were to get suicide thoughts.

**Results:**

The group of people who would not seek help or information for suicide thoughts, were more likely to be male, aged between 40 and 49 years, not being retired, or having experienced suicide or suicide attempts among family or friends, compared to the group that stated their help-seeking intentions. Moreover, they were more likely to report more negative attitudes to suicide (e.g., believing it can’t be prevented, or that it should not be talked about), mental illness (e.g., wanting to hide depression, or wanting to sort out problems on their own), and help-seeking (e.g., feeling inferior if seeking professional help, or not being confident in getting help) as well as lower levels of support from family or friends. Stepwise logistic regression analyses identified male sex, not being confident in getting professional help for suicide thoughts, not being able to talk to family or friends about problems, and not wanting to disclose having depression, as the statistically significant predictors of not wanting to seek help or information, explaining 28% of the variance.

**Conclusions:**

We conclude that these factors should be targeted in future suicide prevention efforts in Norway. In particular, the strongest predictor (Not being confident in getting professional care for suicide thoughts), should be addressed.

**Clinical trial number:**

Not applicable.

## Introduction

Suicide rates in Norway have been largely stable the past three decades, with approximately 650 individuals (12.4 per 100 000) taking their own life annually, about 70% of whom are men [1]. Moreover, the median age of death by suicide in Norway is 47 years, with men significantly outnumbering women in all adult age groups [1]. Mental illnesses, in particular depressive disorders, are strong predictors of suicide [2]. However, about half of all individuals who die by suicide in Norway do not seek contact with specialist mental health services during the year prior to death [3]. Among the people who would not seek help, there are significantly more men than women [3].

Endorsing opinions about suicide that are detrimental to help-seeking, having prejudiced attitudes or stigma towards people with mental illness, and poor general mental health literacy are powerful barriers to seeking professional help for mental health problems in general [4, 5] and for depression and suicide thoughts in particular [6]. These opinions or attitudes may be expressed as statements such as*“When a person has decided to take their life*,* it can’t be prevented”. “If I had depression*,* I would not tell anyone”*,* or “If I were to experience a serious emotional crisis*,* I would not be confident of getting good professional help*”. Carrying self-stigma, such as believing that *“I would feel inferior if I asked a mental health counselor for help”*, is yet another barrier to help-seeking [7–9].

Several demographic factors have been associated with such attitudes and assumptions. In males, they seem to be particularly frequent, perhaps being linked to traditional masculine values of self-reliance and unwillingness to talk about health problems [10–12]. In both younger (< 35 years) [6] and older (> 60 years) [8] individuals, increased levels of opinions that are likely to impede help-seeking for mental ill health or suicide thoughts have been found. Moreover, having experienced suicide or attempted suicide among relatives or friends have been identified as a risk factor for suicide or suicide thoughts in the bereaved [13, 14]. One explanation may be a normalization of suicide that these experiences confer [15]. On the other hand, Han et al. [4] found that losing a family member to suicide increased help-seeking in the bereaved, suggesting that the relationship between suicide exposure and attitudes to help-seeking is not clear-cut.

Attempts have been made to alter attitudes that are associated with lower willingness to seek help for mental health problems and/or suicide thoughts through suicide prevention media campaigns [16–18]. Typically, a campaign consists of information about how to seek help for one’s own suicide thoughts or those of family or friends. These campaigns are usually intended for the entire population that is exposed to them but may be a good way of reaching those who experience suicide thoughts without being in contact with the health care services. In 2022 and 2023, the Norwegian National Centre for Suicide Research and Prevention in cooperation with the Norwegian Directorate of Health conducted suicide prevention media campaigns directed at the general population in two regions– Mid-Norway and West-Norway [16]. Prior to the campaigns, a survey was conducted where attitudes to suicide, mental illness, and help-seeking were assessed in a sample of the population that was later exposed to the campaign message. Among these respondents, a small group stated that they would not seek help or information if they were to experience suicide thoughts. In order to design and implement further suicide prevention interventions in Norway, we believe there is a need for an elucidation of the factors behind these non-help-seeking intentions.

It is well known that attitudes and intentions are strongly related to actual health-promoting behavior in general [19]. There is a small number of studies of how demographic and attitudinal variables explain why some people do not seek help for suicide thoughts. Han et al. [4] and Calear et al. [20] have identified high self-reliance, low suicide literacy, high levels of suicide stigma, and negative attitudes towards help-seeking as important explanatory variables. In a study of mainly female participants, Batterham et al. [21] found that individuals who did not perceive having a mental disorder or being sufficiently distressed were reluctant to contact health care services for suicide thoughts. However, there is a want of studies of attitudes to suicide thoughts and help-seeking intentions in non-clinical, sex- balanced community samples.

In this study, we use a population-drawn sample stratified for sex and age. Our first aim was to compare the demographic characteristics, experiences with suicide behavior among family and friends, and attitudes to suicide and mental illness in the group that would not intend to seek help or information for suicide thoughts and the group that would intend to seek help or information. Second, given the frequently occurring associations between being male and experiencing barriers to seeking help, we aimed to perform subgroup analyses of differences between men who would intend to seek help or information and men who would not. Third, we aimed to identify predictors of not wanting to seek help or information for suicide thoughts.

## Methods

### Participants

The Mid-Norway region includes two counties with 74 local municipalities and a population of approximately 700 000. The West-Norway region includes two counties with 66 local municipalities and a population of approximately 1 100 000 individuals. Both regions consist of larger cities, smaller towns, and rural areas.

The participants in the current study were sampled by the Norstat company (which specializes in Norwegian population and marketing surveys) from their web panel of regular respondents randomly selected from the general population. Inclusion criteria for participation were being aged 18 years or above and being able to fill in digital questionnaires in Norwegian on computers, tablets or smart phones. The sample was stratified according to age, sex, and geographical location (ensuring representation from all regional counties) in order to achieve a balanced panel of respondents. See Table 1 for demographic characteristics.

**Table 1:** Demographic characteristics of the sample (*N* = 3251)

	Non-help-seeking (*n* = 167)	Help-seeking (*n* = 3084)	Test statistics
Sex	106 m (63.5%) 61 f (36.5%)	1535 m (49.8%) 1549 f (50.2%)	X^2^ = 11.90 *p* <.001
Age, years	45.2 (15.8)	48.6 (17.6)	Z = -2.48 p 0.013
Age, median years	45	48	
Age group:			X^2^ = 5.71 p 0.017
< 30	35 (21.0%)	582 (18.9%)	
30–39	22 (13.2%)	509 (16.5%)	
40–49	47 (28.1%)	510 (16.5%)	
50–59	33 (19.8%)	558 (18.1%)	
60–69	18 (10.8%)	449 (14.6%)	
70–79	11 (6.6%)	412 (13.4%)	
80–94	1 (0.6%)	64 (2.1%)	
Education level:			X^2^ = 5.76 p 0.330
Elementary school	12 (7.2%)	126 (4.1%)	
Senior high school	35 (21.0%)	652 (21.1%)	
Senior high school +	39 (23.4%)	624 (20.2%)	
BA	39 (23.4%)	846 (27.4%)	
MA	39 (23.4%)	791 (25.6%)	
Other	3 (1.8%)	45 (1.5%)	
Employment status:			X^2^ = 16.25 p 0.025
Student	17 (10.5%)	287 (9.4%)	
Full or part time work	103 (63.6%)	1766 (57.9%)	
Military service	0	5 (0.2%)	
Parental leave	1 (0.6%)	20 (0.7%)	
Homemaker	4 (2.5%)	26 (0.9%)	
Unemployed	3 (1.9%)	45 (1.5%)	
Disability pension	17 (10.5%)	242 (7.9%)	
Retired	17 (10.5%)	659 (21.6%)	
Mean income level *	5.7 (2.7)	5.8 (2.6)	Z = -1.18 p 0.237
Number of individuals in household	2.5 (1.3)	2.4 (1.2)	Z = -0.12 p 0.908
Attempted suicide among family/friends	80 (47.9%)	1041 (33.8%)	X^2^ = 14.04 *p* <.001
Suicide among family/friends	63 (37.7%)	812 (26.6%)	X^2^ = 10.29 p 0.006

Numbers in individual count (%) or mean (SD), all analyses are non-parametric. Some of the percentages do not add up due to random missing data in Education level and Employment status. *: Likert scale numbers of annual income, 1 = 100 000 NOK to 14 = 1 400 000 + NOK. Test statistics: Chi^2^ test for dichotomous variables, Mann-Whitney U test for the continuous variables

### Survey

This study is part of a large national project conducting public suicide prevention media campaigns in all five Norwegian geographical regions and assessing attitudes to suicide, mental illness, and help-seeking in the population that has been exposed to the campaigns. This work is financed by the Norwegian Directorate of Health, with the National Centre for Suicide Research and Prevention of the University of Oslo as the main academic partner cooperating in developing the campaign material and surveys. Before and after each campaign, the members of the Norstat web panel of the relevant geographical region are invited to fill in a survey (see below, *Independent variables*). This study uses data obtained from the pre-campaign surveys in Mid- and West-Norway. The respondents had not been exposed to the campaign material at that point. In Mid-Norway, 7479 members of the Norstat panel were sent a survey in week 41 of 2022. The survey was completed by 1751 individuals, yielding a response rate of 23%. In West-Norway, 7847 members of the Norstat panel were sent a similar survey in weeks 19–20 of 2023, and 1500 individuals completed it, yielding a response rate of 19%. Thus, the total number of respondents included in this study was 3251. The survey was filled in on computers, tablets, or mobile phones.

### Independent variables

In the survey, the participants provided information on demographic, social, and economic characteristics as well as their previous experience with suicide or suicide attempts among family and friends. Moreover, the following items assessed attitudes towards suicide and mental illness (see Table 2 for item wordings): One item was taken from the Depression Stigma Scale (DSS) [22] where responses were made on a five-point Likert scale from 1 (“Completely disagree”) to 5 (“Completely agree”). Five items were taken from the Attitude to Suicide Scale (ATTS) [23], where responses were made on a five-point Likert scale from 1 (“Completely disagree”) to 5 (“Completely agree”). One item was taken from the Attitudes Toward Seeking Professional Help scale (ATSPH) [24], where response were made on a four-point Likert scale from 1 (“Disagree”) to 4 (“Agree”). Five items were taken from the Self-stigma of Seeking Help scale (SSOSH) [25] with responses made on a five-point Likert scale from 1 (“Completely disagree”) to 5 (“Completely agree”). Two items were taken from the Multidimensional Scale of Perceived Social Support (MSPSC) [26] with response made on a five-point Likert scale from 1 (“Completely disagree”) to 5 (“Completely agree”) and combined into one item. In addition, we constructed one item on confidence in getting good health care with responses made on a five-point Likert scale from 1 (“Completely disagree”) to 5 (“Completely agree”). Two items were questions about the history of attempted suicide or suicide among family and friends, where answers were given as “Yes” or “No” (Tables 2 and 3). The survey items used in connection with the regional campaigns are not identical. For the current paper, the 14 overlapping items of the Mid- and West-Norway surveys were analyzed.

**Table 2:** Attitudes to suicide and help-seeking, entire sample (*N* = 3251)

	Non-help-seeking (*n* = 167)	Help-seeking (*n* = 3084)	Test statistics Mann-Whitney U	Effect size *r*
**Attitudes to suicide**				
I am prepared to help a suicidal person by contacting/talking to him/her	3.9 (1.0)	4.1 (0.9)	-2.10 p.036	-0.04
When a person has decided to take their life, it can’t be prevented	2.4 (1.1)	2.0 (1.0)	-5.30 *p* <.001	-0.09
Suicide is one’s own business that others should not interfere with	2.0 (1.0)	1.6 (0.8)	-5.74 *p* <.001	-0.10
There is a risk of evoking suicide thoughts if one asks about it	2.9 (1.1)	2.6 (1.0)	-2.62 p 0.009	-0.05
Suicide is a topic that should not be discussed	2.0 (1.1)	1.7 (0.9)	-3.03 p 0.002	-0.05
If I had suicide thoughts, I am confident of getting good health care	2.2 (1.1)	3.3 (1.2)	-10.82 *p* <.001	-0.19
**Attitudes to help-seeking for mental ill health**				
If I had depression, I would not tell anyone	3.9 (1.1)	2.7 (1.1)	-11.88 *p* <.001	-0.21
People should work out their own problems, getting psychological help should be the last resort	1.8 (0.9)	1.6 (0.9)	-2.53 p 0.012	-0.04
**Self-stigma towards help-seeking for mental ill health**				
If I sought professional help I would be less satisfied with myself	2.8 (1.2)	2.1 (1.0)	-7.02 *p* <.001	-0.12
My self-esteem would NOT be threatened if I sought professional help	3.0 (1.3)	3.9 (1.1)	-8.60 *p* <.001	-0.15
I would feel inferior if I asked a mental help counselor for help	2.8 (1.3)	2.1 (1.1)	-6.35 *p* <.001	-0.11
I would have negative feelings for myself if I couldn’t solve my own problems	3.6 (1.1)	2.9 (1.2)	-7.51 *p* <.001	-0.13
I would feel OK deciding to seek out professional help	3.1 (1.2)	4.1 (0.9)	-11.01 *p* <.001	-0.19
**Social support**				
I can talk to my friends or family about my problems	2.7 (1.2)	3.7 (0.9)	-10.96 *p* <.001	-0.19

Numbers in mean (SD)

**Table 3:** Attitudes to suicide and help-seeking, males (*N* = 1641)

	Non-help-seeking (*n* = 106)	Help-seeking (*n* = 1535)	Test statistics Mann-Whitney U	Effect size *r*
**Attitudes to suicide**				
I am prepared to help a suicidal person by contacting/talking to him/her	3.9 (1.1)	4.1 (1.0)	-1.46 p.145	-0.04
When a person has decided to take their life, it can’t be prevented	2.4 (1.0)	2.0 (1.0)	-4.22 *p* <.001	-0.10
Suicide is one’s own business that others should not interfere with	2.1 (1.0)	1.7 (0.9)	-3.99 *p* <.001	-0.10
There is a risk of evoking suicide thoughts if one asks about it	3.0 (1.0)	2.8 (1.0)	-1.71 p 0.088	-0.04
Suicide is a topic that should not be discussed	2.1 (1.0)	1.9 (1.0)	-1.61 p 0.108	-0.04
If I had suicide thoughts, I am confident of getting good health care	2.3 (1.0)	3.4 (1.1)	-8.83 *p* <.001	-0.22
**Attitudes to help-seeking for mental ill health**				
If I had depression, I would not tell anyone	4.0 (1.1)	2.8 (1.1)	-9.20 *p* <.001	-0.23
People should work out their own problems, getting psychological help should be the last resort	2.0 (1.0)	1.8 (0.9)	-2.22 p 0.027	-0.05
**Self-stigma towards help-seeking for mental ill health**				
If I sought professional help I would be less satisfied with myself	2.9 (1.3)	2.3 (1.1)	-5.11 *p* <.001	-0.13
My self-esteem would NOT be threatened if I sought professional help	3.0 (1.3)	3.8 (1.1)	-6.34 *p* <.001	-0.16
I would feel inferior if I asked a mental help counselor for help	2.8 (1.3)	2.3 (1.1)	-4.30 *p* <.001	-0.11
I would have negative feelings for myself if I couldn’t solve my own problems	3.6 (1.1)	3.0 (1.1)	-4.87 *p* <.001	-0.12
I would feel OK deciding to seek out professional help	3.1 (1.2)	4.0 (1.0)	-7.85 *p* <.001	-0.19
**Social support**				
I can talk to my friends or family about my problems	2.8 (1.2)	3.7 (1.0)	-7.45 *p* <.001	-0.19

Numbers in mean (SD)

Using the questionnaires and scales described above in their entirety would be ideal and provide a nearly complete picture of the respondents’ attitudes we wish to study. However, this would render the surveys very long, risking a lower response rate. Therefore, for the regional campaigns, we selected the questionnaire items tapping attitudes that we decided were the most important to change through the campaigns. The selection was based, firstly, on findings from relevant studies in this field, in which the most important attitude barriers to help-seeking for suicide thoughts and depression were identified [8, 9]. Secondly, the current campaigns were developed in cooperation with the Norwegian Directorate of Health. Since 2019, this institution has monitored the attitudes to suicide in the Norwegian population through annual surveys of the entire nation. In order to prepare for a future comprehensive project on long-term changes in attitudes to suicide in Norway, and the effects of regional campaigns on these attitudes, it was determined to use the same items in the current regional campaign surveys as in the national monitoring project.

### Dependent variable

The dependent variable was based on the question “If you had suicide thoughts, where would you seek help or information?”. This question was added to the campaign surveys because it is a fixed feature of the national monitoring project of the Directorate of Health, and a list of places to seek help (e.g., family, friends, colleagues, general practitioner, emergency services, websites, telephone hotlines etc.) was provided (data not shown). The respondents selected the place(s) that they found suitable. The response to the final alternative, “I would not seek help or information” was the dependent variable of this study. A small group of people replied that “I would not seek help or information” (*n* = 167, 5.1% of the sample), and a much larger group stated that they would seek help from one of the sources listed above (*n* = 3084). In this paper, the first group is described as “non-help-seeking” and the second group as “help-seeking”.

### Ethical approval

All respondents had given their broad, informed consent to membership of the Norstat web panel. This broad consent implies the right to refrain from participation in surveys without having to state a reason for declining. This project was carried out in keeping with the principles of the Helsinki Declaration.

### Statistics

Due to the large difference in sample sizes, non-parametric Mann-Whitney U-tests for group comparisons were used for the main analyses in Tables 1, 2 and 3. Effect sizes are given as Pearson’s r, with the cutoffs for effect magnitude being 0.1 (low), 0.3 (medium), and 0.5 (large). For the analyses of sex differences in Supplemetary Table 1, Student’s t-tests for independent samples were used with Cohen’s d indicating effect sizes, with the cutoffs for effect magnitude being 0.2 (small), 0.4 (medium), and 0.6 (large).

For the logistical regression analyses, the dependent variable was binary and was based on either wanting to or not wanting to seek help or information for suicide thoughts. In order to identify predictors of not wanting to seek help, a stepwise forward LR logistic regression analysis was run with age groups and sex in the first step and all the remaining demographic and attitude variables in the second step. The independent variables that showed a statistically significant association with the dependent variable were then selected for the final analyses (Tables 4 and 5).

**Table 4:** Logistic regression analysis results, all models (*N* = 3251)

Model	β	Wald	Exp(β)	CI Exp(β)	*R* ^2^
1: Male sex Age groups I can talk to my friends or family about my problems	0.512 − 0.121 − 0.897	8.15 ** 5.40 * 129.87 ***	1.67 0.88 0.41	1.17–2.37 0.80–0.98 0.35–0.48	0.15
2: Model 1 + If I had suicide thoughts, I am confident of getting good health care	− 0.672	61.58 ***	0.51	0.43–0.60	0.22
3: Model 2 + If I had depression, I would not tell anyone	0.600	39.45 ***	0.54	0.46–0.64	0.26
4: Model 3 + I would feel OK deciding to seek out professional help	− 0.350	17.40 ***	0.70	0.60–0.83	0.27
5: Model 4 + When a person has decided to take their life, it can’t be prevented	0.312	14.17 ***	1.37	1.16–1.61	0.28

Model: Independent variable(s). R^2^: Nagelkerke. *: *p* <.05, **: *p* <.01, ***: *p* <.001

Dependent variable: “I would not seek help or information if I had suicide thoughts”

**Table 5:** Logistic regression analysis results, model 5 (*N* = 3251)

Independent variables	β	Wald	Exp(β)	CI Exp(β)
Male sex	0.511	7.22 **	1.67	1.15–2.41
Age group	− 0.020	0.13	0.98	0.87–1.10
When a person has decided to take their life, it can’t be prevented	0.312	14.17 ***	1.37	1.16–1.61
If I had suicide thoughts, I am confident of getting good health care	− 0.582	44.62 ***	0.56	0.47–0.66
If I had depression, I would not tell anyone	0.486	24.54 ***	1.63	1.34–1.97
I would feel OK deciding to seek out professional help	− 0.342	16.38 ***	0.71	0.60–0.84
I can talk to my friends or family about my problems	− 0.436	23.34 ***	0.65	0.54–0.77

**: *p* <.01, ***: *p* <.001; Dependent variable: “I would not seek help or information if I had suicide thoughts”

Logistic regression is appropriate when the smallest group is > 1% the size of the total sample. Our smallest group is > 5% the size of the total sample, therefore our uneven group size is of no concern for this analysis.

## Results

### Group differences in intentions to seek help or information

The group of people who would not seek help or information for suicide thoughts (*n* = 167), were more likely to be male, aged between 40 and 49 years, not being retired, and having experienced suicide or suicide attempts among family or friends (Table 1). Moreover, they were more likely to report more negative attitudes to suicide, mental illness, and help-seeking, as well as lower levels of support from family or friends (Table 2). Then, we compared the attitudes to suicide, mental illness, and help-seeking in the males who stated that they would not seek help or information for suicide thoughts (*n* = 106) and the males who stated that they would (*n* = 1535) (Table 3). The result of this sub-group analysis of men revealed a nearly identical pattern of results as for the entire sample, in that non-help-seeking intention was associated with more negative attitudes to suicide, mental illness, help-seeking, and low levels of support from family and friends. The effect sizes reported in Tables 2 and 3 are low, however, despite most group differences being statistically significant.

In order to investigate possible sex differences in attitudes to suicide, mental illness, and help-seeking, we caried out independent-samples analyses comparing the responses of men and women. The results revealed that for 11 of the 14 items, men displayed significantly less suicide literacy and willingness to disclose mental problems, and more stigma towards mental illness. Men were, however, significantly more confident of getting good health care compared to women (Supplementary Table 1). The effect sizes were low to medium.

### Predictors of not wanting to seek help or information

The stepwise forward logistic regression analysis generated five models. The final model, explaining 28% of the variance, consisted of seven predictors, of which six were statistically significant; age group remained non-significant in all models. The first three models explained most of the variance and consisted of the following significant predictors: male sex, not being able to talk to family or friends about problems, not being confident in getting professional help for suicide thoughts, and not wanting to disclose having depression (Tables 4 and 5).

## Discussion

### Group differences in intentions to seek help or information

In this population-based study, we investigated the demographic characteristics and attitudes to suicide and help-seeking for suicide thoughts and mental illness in individuals who stated they would seek help or information versus those who would not seek help or information for suicide thoughts. There were quite distinct and relatively consistent group differences, both in the analyses of the entire sample and in the male versus female subgroups. When interpreting the results, it is necessary to bear in mind that in general, the group differences were small, although statistically significant. The effect sizes were small, and the differences in responses to the survey items were usually less than 1 Likert scale point. Most of the responses seemed to cluster around the scale point 3, which indicates neutrality towards the attitude expressed or the question asked.

The first major finding is that the group of respondents who would not seek help or information consisted of significantly more males and individuals between 40 and 49 years of age, and a significantly higher number who reported experiences with suicide or attempted suicide among family or friends. These results are consistent with some previous research. In Norway, a very high proportion of those who die by suicide are men between 40 and 59 years of age [1]. Others have found that non-help-seekers tend to be young adults, even when experiencing significant psychological distress due to suicide thoughts [27–29]. Thus, the relationship between age and help-seeking may not be linear.

Moreover, being exposed to suicide or suicide attempts among family or friends is a risk factor for one’s own suicide behavior [13, 14]. For some of these individuals, suicide among close family or friends may normalize suicide to the extent that would diminish help-seeking if one were to experience suicide thoughts [15]. On the other hand, it has been pointed out that exposure to suicide may not be inherently risky, as watching how such tragedies affect others may motivate towards help-seeking [4, 29]. Moreover, the circumstances of suicide are probably important factors impacting the way the bereaved deal with their loss. If a close family member or friend dies by suicide, the pain of the bereaved is probably stronger than if a relatively remote friend dies. Other relevant factors may be the age and the mental health of the bereaved, their closeness to the scene of the suicide, and the degree of support they receive from others. It is a limitation of the present study that we did not separate between experiences with suicide or suicide attempts in family members versus friends.

The group with no intentions to seek help or information reported several instances of negative attitudes to suicide and help-seeking for suicide thoughts or emotional problems, social or emotional isolation, and self-stigma in the face of own mental problems. This is consistent with the findings of others [4, 9, 20]. This pattern of results was mostly sustained in the subgroup analysis of men only. The small group of men who stated that they would not seek help or information for suicide thoughts was characterized by the same self-stigma and negative attitudes to help-seeking as the entire non-help-seeking group. However, the male pattern of response was not entirely detrimental to help-seeking, as there were three attitudes that were not significantly different between the male groups. Thus, not wanting to seek help or information for suicide thoughts is not a universal attitude among men.

### Predictors of not wanting to seek help or information

Our second major finding was the identification of factors that related most strongly to not wanting to seek help or information for suicide thoughts. We found that those were male sex, not being able to talk to close ones about problems, lacking confidence in health care services, or having negative attitudes to depression and help-seeking for mental illness. Together, these variables explained 28% of the variance in willingness to seek help.

Model 1 of the regression analysis, containing half of the level of explained variance, consisted of male gender and not being able to talk to family and friends about problems. In Norway, the male suicide rate is higher than the female across all socioeconomic strata [30, 31]. A reluctance among men to seek professional help for suicide thoughts or emotional problems that predict suicide behavior is an important explanatory factor [32, 33]. This underutilization of health care services may have multiple causes. Most studies point to traditional masculine norms, e.g., self-reliance, stoicism, and need for emotional control, as significantly deterring men from seeking help for suicide thoughts [23, 33] or psychological problems with strong links to suicide behavior [10, 11]. As we did not measure the presence of masculine norms in our study sample, we have no way of determining whether such motivators accounted for their barriers to help-seeking. However, some of the items of our questionnaire may tap traits of self-reliance and stoicism, for example the statement “People should work out their own problems, getting psychological help should be the last resort”. In this respect, we may indirectly have measured masculine norms. Moreover, traditional masculine norms are likely to have influenced the responses in our sample, as they have consistently been found to affect attitudes to psychological treatment and help-seeking across countries and age groups [10]. This suggestion is strengthened by our finding of statistically significant sex differences in our participants in nearly all the items measuring suicide literacy and attitudes to help-seeking.

The next important predictor variable of not wanting to seek help or information for suicide thoughts was disagreement with the statement “I can talk to my friends or family about my problems”. The absence or presence of a social network may influence attitudes and help-seeking in several and sometimes opposite ways.

Not having friends or family, or the inability to rely on existing friends or family for emotional support, are known to increase risk of suicide, particularly in people with depression [34]. Moreover, social or emotional isolation may be predictive of non-help-seeking intentions [4]. Possibly, isolation or loneliness are detrimental to mental health literacy, in that lonely individuals have not learnt to recognize symptoms of psychological distress, how to manage them, and where to seek help.

The presence of reliable family or friends is usually assumed to have beneficial practical and emotional effects. The care from significant others may both diminish the need for help from other sources, or network members may identify problems that need professional treatment and encourage health care service utilization [4]. However, in some cases, family and friends may foster stigma and negative attitudes to help-seeking if they have these themselves. This seems particularly relevant in light of the above discussion of traditional masculine norms [10].

The next significant predictor, “If I had suicide thoughts, I am confident of getting good health care”, was the independent variable with the strongest contribution to the outcome variable. Disagreement with the above statement was predictive of not wanting to seek help or information for suicide thoughts, suggesting that lack of confidence in the health care services fosters unwillingness to seek help. This is logical and is particularly striking when taken together with the finding that there was a significantly larger proportion of individuals who had experienced attempted suicide or suicide among family and friends in the non-help-seeking group. One possible explanation may be the normalization of suicide that exposure may bring about [15]. Alternatively, fear of the consequences of disclosing suicide thoughts to health care professionals may be important. Reasons for this include fear of upsetting the clinician by talking about a highly emotionally charged topic, fear of being (forcibly) hospitalized, shame of having suicide thoughts, or the inability to find the words to talk about this difficult topic [35].

In addition, the group that would not seek help or information for suicide thoughts may have observed close ones not getting proper help for their struggles, and lost confidence in society’s ability to help. In light of Norwegian mental health care being a severely underfunded sector with long waiting lists compared to the rest of the national public health care service, this topic remains a possibility, although a remote one.

Agreement with the statement “If I had depression, I would not tell anyone” was the next significant predictor of not wanting to seek help or information for suicide thoughts. This statement may reflect a negative attitude to depression, suggesting that mental illness confers stigma or inferiority status on the person experiencing it. Negative attitudes to the problem in question is well known as a barrier to help-seeking [5, 11]. In men in particular, the association between negative attitudes to psychological distress and help-seeking is strong [12]. However, talking about having depression requires the presence of other people. Individuals without family or friends may simply lack the opportunity to talk. In this respect, withholding depression may be motivated by the same factors that were discussed above concerning the predictor “I can talk to my family and friends about my problems”.

Disagreement with the statement “I would feel OK deciding to seek out professional help” was another significant predictor of the outcome variable, suggestive of a self-stigmatizing attitude to asking for help from the health care services. Stigmatizing attitudes have been identified as explanatory factors in delaying or refusal of help-seeking, especially in men [5]. On the other hand, as was suggested above, it is possible that previous experiences of not getting the help they or someone close to them need may generate a feeling of professional help-seeking being futile. Therefore, not feeling OK deciding to seek out professional help is not necessarily the result of stigmatizing attitudes to mental health care, but of unfortunate experiences with care providers.

Low suicide literacy (i.e., lack of knowledge about suicide and/or belief in myths about suicide) has been associated with negative attitudes to help-seeking for own suicide thoughts [20]. Belief in suicide myths have also been found to deter helping others who express suicide thoughts or psychological distress [8]. One item assessing this factor in our study was the statement “When a person has decided to take their life, it can’t be prevented”. Endorsement of this statement was significantly related to not wanting to seek help or information in our sample.

In summary, we have identified a set of variables that accounted for a moderate amount of variance in not wanting to seek help or information for own suicide thoughts. Variables such as being male, lacking social support, low confidence in the ability to get help, stigmatizing attitudes to mental illness, and low suicide literacy were key factors. Bearing in mind that the level of explained variance was moderate, and that the cross-sectional nature of our study prevents us from determining the causal or temporal relationship between these factors, we aim to target these variables in future suicide prevention media campaigns in Norway.

### Strengths and limitations

The major strengths of this study were our population-based sample stratified according to sex and age. Second, we use the same questionnaire items as the annual Norwegian national monitoring of attitudes to suicide project, enabling us in the future to chart long-term trajectories of suicide literacy and help-seeking attitudes.

The major limitations are first our low number of individuals (*N* = 167) who would not seek help or information for suicide thoughts. This is the group of people that we must reach if we are to increase help-seeking and characterizing them in as much detail as possible is pivotal. However, the low N renders us unable to perform subgroup analyses, e.g., separate analyses of attitudes to help-seeking in each age group. A second limitation is that we have asked about intentions for help-seeking or information only, and not measured actual behavior, such as visits to health care services for suicide thoughts or symptoms of mental illness.

Third, we have analyzed independent associations with the dependent variable, and have no information about how the independent variables cluster together in each individual. In the future, the interrelations between some of the variables relevant for targeted interventions against suicide should be explored. One example is the need for determining the direction of the relationship between the statements “If I had depression, I would not tell anyone” and “If I had suicide thoughts, I am confident of getting good health care”. In our study, agreement with the first statement and disagreement with the second was associated with not wanting to seek help or information for suicide thoughts. These attitudes could be dismissed as being due to self-stigma or unfounded prejudice but could also result from actual negative experiences with help-seeking.

Fourth, we had no information about the respondents’ previous experiences of mental ill health or use of mental health services, as it has been demonstrated that personal experience of depression or suicide thoughts may be both a barrier to help-seeking [9, 12, 36] or a motivation factor [4, 29]. Moreover, we did not have information pertaining to treatment access in the respondents. In rural areas health care facilities are usually few and far between, and in urban areas the mental health treatment clinics have long waiting lists. This issue is particularly important to investigate further given the strong predictive power of the variable ““If I had suicide thoughts, I am confident of getting good health care”.

Fifth, the respondents to the survey were self-selected, although sampled for sex and age representativity by the polling agency. In addition, the response rate was ~ 20%. These factors limit the generalizability of our results.

Sixth, the dependent variable contained a dual message that may have generated confusion in the respondents. We asked if they would seek “help or information” for suicide thoughts, and asking about help or information in two separate questions would have been better and enabled us to draw clearer conclusions. Moreover, different individuals may understand the concept of “suicide thoughts” differently. For some, these are fleeting and mild, for others persistent and severe. Our study is limited by our not providing clear definitions of the nature of suicidal ideation.

The final limitation concerns the selection of independent variables assessing attitudes to suicide, mental illness, and help-seeking. We selected the questionnaire items tapping attitudes that we decided were the most important to change through the Norwegian suicide prevention campaigns. Importantly, these items are used in the annual national monitoring of the attitudes to suicide. By using only a few items from relevant questionnaires and scales we are left with insufficient information about the details of attitudes to suicide and help-seeking, but we generate data that will be used for large, nation-wide studies of changes in these variables.

Relatedly, this study was not theory-driven. In order to understand human behavior, it is recommended to use a theoretical foundation for the relationship between attitudes, intentions, and practical actions, such as help-seeking. For reasons already mentioned, the present selection of survey items did not permit a theory-driven investigation. However, we are in the process of carrying out regional suicide prevention projects using the Theory of Planned Behavior [19] as a basis.

### Practical implications

Suicide prevention efforts take many forms, such as population-based media campaigns encouraging people to seek help for suicide thoughts or mental ill health, training of GPs to recognize signs of patients’ having suicide thoughts or allocating more financial resources to mental health professionals caring for people with mental illness. However, all of these strategies require people to perform the act of seeking help or information when facing a crisis. Reaching the individuals who do not intend to seek help may require different methods than those directed at people with suicide thoughts or mental ill health who want to seek help. We are unsure about how to reach the former group. The results of the present study suggest, however, that some members of the general population have vulnerability linked to attitudes or social experiences that may impede their help-seeking behavior. This vulnerability consists mainly of lack of trust in the ability to get professional help as well as social or emotional isolation. A first step in the direction of increasing help-seeking would be to target these areas directly in future suicide prevention strategies.

Apart from reducing or removing the methodological limitations outlined above, a way forward would be to target male members of the population specifically. Simultaneously, increasing confidence in mental help services and reducing stigma of help-seeking are other focus areas. Providing clear advice about how to talk to friends and family about suicide thoughts and emotional problems is yet a necessary future intervention, as is encouraging the use of telephone helplines, chat or messaging services for those without social networks.

## Data Availability

The data that support the findings of this study are available from Norstat but restrictions apply to the availability of these data, which were used under license for the current study, and so are not publicly available. Data are however available from the authors upon reasonable request and with permission from Norstat.
